# Integration of Non-Thermal Plasma into the Thyroidectomy Surgical Workflow: A Prospective Pilot Study on Early Postoperative Safety and Tissue Recovery

**DOI:** 10.3390/biomedicines14102302

**Published:** 2026-10-10

**Authors:** César Jaramillo-Martínez, Régulo López-Callejas, Mario Betancourt-Ángeles, Alberto Martínez-Martínez, Benjamín Gonzalo Rodríguez-Méndez, Diego Medina-Castro, Antonio Mercado-Cabrera, Raúl Valencia-Alvarado, Rosendo Peña-Eguiluz

**Affiliations:** 1 Instituto de Seguridad Social del Estado de México y Municipios (ISSEMYM) Medical Centre Toluca, Av. Baja velocidad 284 km. 57.5, San Jerónimo Chicahualco, Metepec 52170, Estado de México, Mexico; cesar.jaramillo@issemym.gob.mx (C.J.-M.); mario.betancourt@issemym.gob.mx (M.B.-Á.); alberto.martinez@udemex.edu.mx (A.M.-M.); 2 National Institute of Nuclear Research, Carretera México Toluca-La Marquesa s/n, Ocoyoacac 52750, Estado de México, Mexico; regulo.lopez@inin.gob.mx (R.L.-C.); benjamin.rodriguez@inin.gob.mx (B.G.R.-M.); antonio.mercado@inin.gob.mx (A.M.-C.); raul.valencia@inin.gob.mx (R.V.-A.); rosendo.eguiluz@inin.gob.mx (R.P.-E.)

**Keywords:** cold atmospheric plasma, non-thermal plasma, thyroidectomy, wound healing, haemostasis

## Abstract

**Background:** Thyroidectomy is a cornerstone treatment for thyroid pathologies but carries risks including recurrent laryngeal nerve injury, hypoparathyroidism, and cervical haematomas. Non-thermal plasma (NTP) may improve local haemostasis and tissue healing, although its safety within the cervical neurovascular axis requires human validation. **Methods:** This prospective, interventional, single-arm pilot study evaluated the feasibility, reproducibility and early postoperative safety of integrating atmospheric-pressure NTP (0.5 W/cm^2^) into the standard thyroidectomy workflow in 30 patients. Safety endpoints included the absence of thermal injury, neurovascular complications, or surgical-site morbidity. We assessed healing and aesthetic outcomes using the Vancouver Scar Scale (VSS) and Patient and Observer Scar Assessment Scale (POSAS). **Results:** NTP integration achieved 100% technical feasibility, without reactor failures, gas-delivery interruptions, or thermal anomalies. No major acute complications occurred, including obstructive haematomas, recurrent laryngeal nerve injuries, or severe postoperative hypocalcaemia (0/30; 0.0%). Early incisional assessments showed favourable scores (median VSS: 1.2, IQR: 1.0–2.0; median POSAS overall opinion: 1.1, IQR: 1.0–1.5), reported descriptively at the 14-day postoperative milestone. Intraoperative biophysical evaluations and postoperative clinical tracking revealed no overt thermal anomalies or deep structural alterations, supporting high biological safety. **Conclusions:** Integration of NTP into the endocrine neck surgery workflow demonstrates technical feasibility, logistical efficiency, and a favourable early postoperative safety profile. The intervention is associated with a favourable early incisional healing profile without compromising adjacent vital structures. These preliminary findings support NTP as a reproducible, reliable surgical adjunct and provide a robust clinical and methodological basis for endocrine surgical practice.

## 1. Introduction

Thyroid pathologies represent a considerable public health challenge, ranging from benign nodules and multinodular goitres that cause compressive symptoms to neoplastic processes whose global incidence has shown a sustained increase [1]. Thyroidectomy, total or partial, remains the treatment of choice for multiple clinical indications, including large symptomatic masses, suspected malignancy, or indeterminate cytology (Bethesda III to V) [2]. Despite improvements in surgical technique, this procedure is associated with risks of critical perioperative morbidity, such as hypoparathyroidism, recurrent laryngeal nerve injury, and surgical-site infections [3,4].

Furthermore, the rapid formation of postoperative cervical haematomas, with a reported incidence of 0.45–4.2%, constitutes one of the most serious acute complications of thyroid surgery because it can cause life-threatening airway obstruction [5,6,7]. In this surgical context, thyroid procedures involve the management of both benign and malignant conditions, including differentiated thyroid cancer [8,9,10,11]. In this context, non-thermal plasma (NTP) has emerged as a potential adjunctive technology for local tissue management during thyroid surgery. Its application is intended to complement, rather than replace, established surgical techniques. Consequently, clinicians need to optimise local tissue handling, facilitate effective haemostasis, and minimise intraoperative risks while preserving adjacent neurovascular and glandular structures.

To complement the surgical bed and reduce complications, researchers have explored ablation alternatives such as radiofrequency, microwave, and laser systems [12,13]. However, because they rely on photothermal mechanisms or molecular friction, they carry the inherent risk of thermal dispersion toward critical cervical neurovascular structures and lack selective cellular biochemical interaction [14,15,16]. In contrast, NTP or cold atmospheric plasma (CAP) operates below 35 °C, allowing tissue interaction without damaging conductive transfer [17]. Through a synergistic arrangement of reactive oxygen and nitrogen species (RONS), NTP induces favourable biological responses, including modulation of inflammation, stimulation of tissue repair, and precise biochemical tissue interactions [18,19,20].

Over the past decade, NTP’s potential in wound healing and infection control has been demonstrated [21,22], and our research group has thoroughly characterised its biocompatibility, tissue kinetics, and electromechanical safety profiles across various clinical settings [23,24,25]. However, evidence regarding its direct application to the neurovascular and glandular cervical axis during thyroidectomy in humans is still emerging.

This pilot study aimed to evaluate the operational feasibility and early postoperative safety of integrating NTP into the standard thyroid surgery workflow. Because the study focuses on technical feasibility and monitoring intraoperative tissue response, it was designed as a prospective cohort study without a control group, allowing detailed observation of the device’s performance in a real-world surgical setting. The results provide fundamental data on safety and the integration of NTP into the surgical bed and lay the clinical groundwork for future randomised controlled trials to validate its efficacy.

## 2. Materials and Methods

### 2.1. Study Design and Ethical Approval

This project used a prospective, interventional, single-arm clinical design. The Research Ethics Committee of the ISSEMYM Medical Centre Toluca reviewed and formally approved the protocol on 13 February 2025 (registration number CISEI-201/25, session 565/2025). Before any surgical procedure, we voluntarily obtained written informed consent from all participants in accordance with the Declaration of Helsinki.

The study was initially conceived and conducted as an institutional quality-assurance and feasibility evaluation of a surgical adjunct within the standard thyroidectomy workflow. While preparing the manuscript for international publication, we identified the need for public international trial registration; consequently, we retrospectively registered the study on ClinicalTrials.gov on 2 February 2026, under the identifier NCT07382973, which maps directly to protocol CISEI-201/25.

### 2.2. Study Population, Sample Size Justification, and Selection Criteria

The target population comprised a prospective cohort of 30 patients scheduled for thyroid surgery at the ISSEMYM Medical Centre Toluca between June 2025 and May 2026, consisting of an even distribution of total thyroidectomies (15 patients, 50.0%) and conservative or partial resections, including hemithyroidectomies, subtotal, or quasi-total thyroidectomies (15 patients, 50.0%). To align with international guidelines for early-stage biomedical innovation and pilot trials of medical devices, we determined the sample size. Following methodological frameworks for exploratory clinical research, we pragmatically set the sample size at 30 participants for this pilot study [26,27]. While this cohort size is standard for assessing initial procedural feasibility, workflow integration, and preliminary variance estimation before larger randomised trials, it is acknowledged that it lacks the statistical power to definitively establish safety profiles for infrequent or rare complications of thyroidectomy. Consequently, the cohort size was designed to ensure rigorous safety monitoring and accurate basic descriptive analysis, without requiring a formal a priori comparative power calculation at this stage of development.

The selection criteria were structured as follows:

- Inclusion criteria: Patients of both sexes; ages between 18 and 75 years; scheduled for total, hemithyroidectomy, subtotal or quasi-total thyroidectomy; and voluntary acceptance through the signing of the informed consent.
- Exclusion criteria: Active infections in the surgical region; history of cervical radiotherapy or chemotherapy; decompensated autoimmune diseases; and implantable electronic devices (pacemakers or defibrillators) due to the potential risk of electromagnetic interference. Additionally, to eliminate bias due to extreme anatomical or haemostatic variability, the following cases were excluded.
  ○ Documented coagulopathy or active haematological disorder (International Normalised Ratio, INR > 1.5 or platelet count <100,000/μL).
  ○ Intrathoracic goitres with mediastinal extension below the sternal notch that would compromise ergonomics or standard surgical access.
  ○ Macroscopic invasion of deep adjacent structures requiring composite airway resection or radical neck dissection.
  ○ History of ipsilateral cervical surgical approaches (reoperations).
- Elimination criteria: Voluntary withdrawal of informed consent at any stage; loss of scheduled clinical follow-up; or the development of an acute obstructive cervical haematoma requiring urgent reintervention. This last event was established as an elimination criterion only for the mid-term tissue follow-up analysis and was mandatorily recorded as a primary adverse event within the NTP safety assessment.

**Figure 1:**
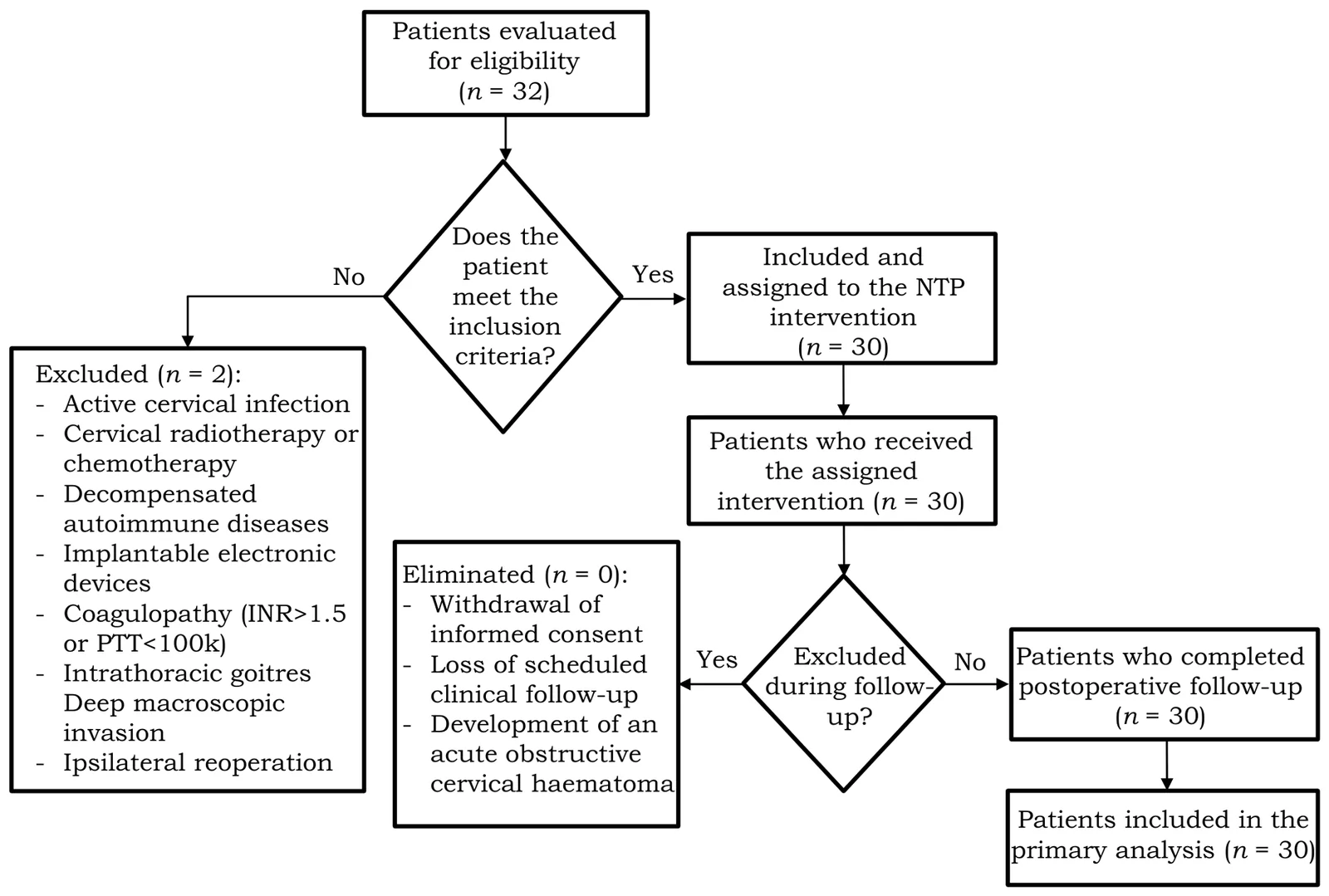
Flow diagram of the study illustrating cohort selection, reasons for pre-intervention exclusion, allocation and receipt of the NTP intervention, and follow-up and inclusion in the final primary analysis.

The flow of participants through each stage of the study, including eligibility screening, pre-intervention exclusions, assignment to the NTP intervention, and follow-up, is detailed in Figure 1.

### 2.3. Experimental Procedure and Operating Parameters

**Figure 2:**
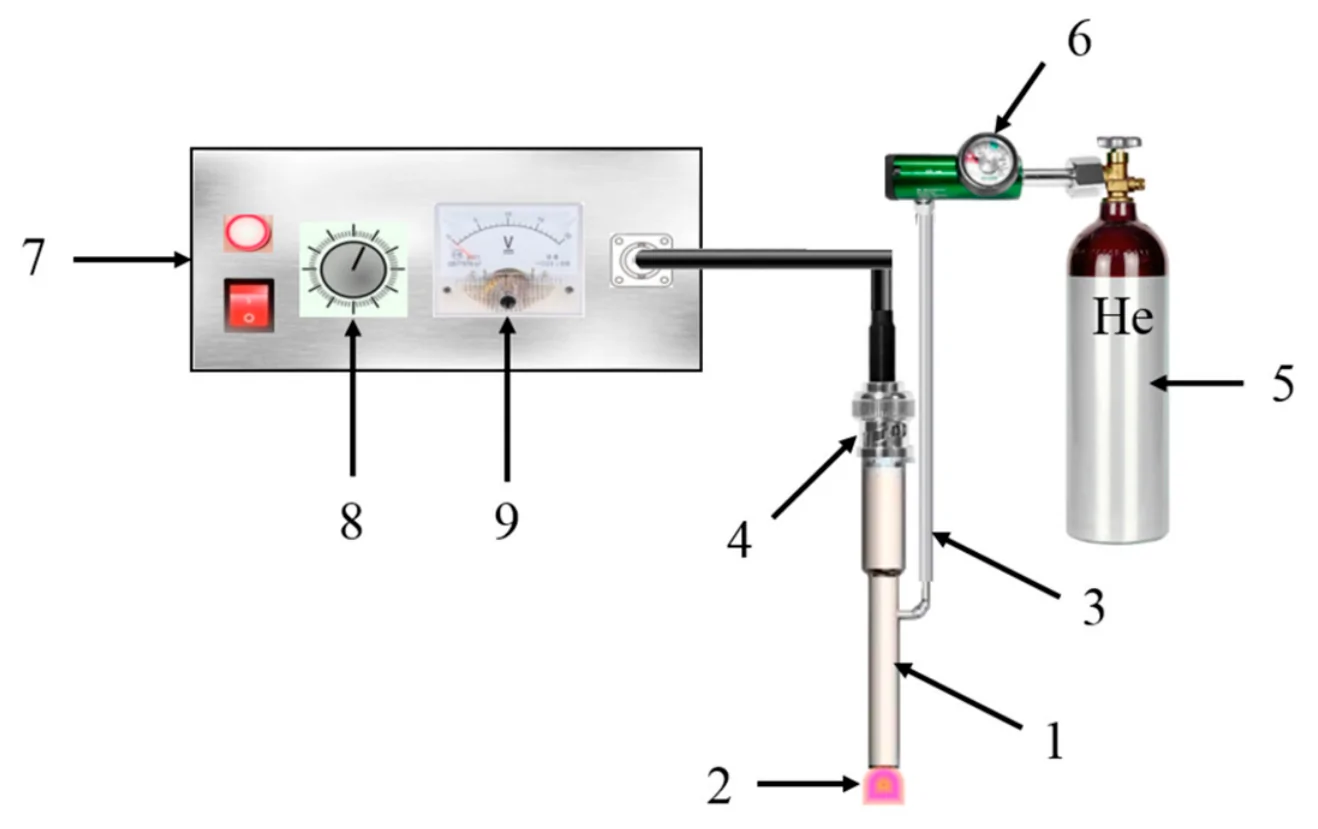
Schematic diagram of the NTP system used in the surgical bed. The system comprises a plasma needle with a dielectric body (1) that generates a plasma plume (2) at its distal end. High-purity helium gas (5) is dispensed through a flow and pressure regulator (6) connected to the gas supply line (3). Power is supplied to the needle via a voltage connector (4) coupled to the main radiofrequency generator (7), which has a power control (8) and an analogue monitoring voltmeter (9).

The experimental procedure involved directly applying NTP to the cervical surgical bed using an atmospheric-pressure plasma needle device. The system operates via electrical excitation in the radiofrequency (RF) spectrum, uses high-purity helium (99.999%) as the discharge gas, and is specifically configured for high-precision clinical interventions. Figure 2 details the reactor’s structural components. The device operated under a constant laminar helium flow regime, strictly regulated at 0.5 litres per minute (LPM) using a high-precision mass flow controller. Ionisation of the carrier gas was achieved through capacitive RF coupling at a standardised centre frequency of 13.56 MHz, with a nominal generator output power of 20 W.

While these operating conditions were designed to yield a maximum estimated tissue power density of 0.5 W/cm^2^ at the treatment surface, we selected these energy-delivery parameters based on validated biophysical models of controlled thermal and electrical energy transfer, optimisation of RONS kinetic fluxes, and a comprehensive analysis of biological safety margins documented in the recent literature [28]. Based on prior diagnostic plasma characterisations and biocompatibility studies by our research group [23,24,25], maintaining a precise, static distance in a dynamic surgical environment remains technically challenging. In practice, the application distance averaged approximately 5 mm. This range was not arbitrary: the spatial and temporal thermal behaviour of this device has been previously characterised [29], showing that when the plasma needle is held stationary, the generated temperature increases steeply at working distances below 3 mm.

For this reason, the clinical protocol mandates a working distance > 3 mm whenever anatomically feasible, along with continuous slow circular displacement of the probe over the treated surface, thereby excluding by design the only condition under which thermal accumulation has been documented. The plasma effluent temperature remained strictly controlled within a safe biological range of 28 to 30 °C, supporting a biological interaction mediated primarily by biophysical processes and RONS kinetics, while minimising the risk of macroscopic thermal damage or structural tissue alteration [30].

To ensure complete procedural reproducibility, we standardised the application protocol across all interventions. For the deep surgical thyroid bed, the plasma needle was applied using a continuous slow circular sweep (~1 cm/s) across the entire area for a total duration of approximately 8 to 10 min, exercising strict anatomical care to avoid direct application over the recurrent laryngeal nerve pathways and applying minimal exposure over the tracheal cartilage. For the exposed prethyroid muscle plane, treatment involved continuous application over approximately 10 to 12 min using the same slow circular displacement, to achieve homogeneous superficial management and stabilisation of the fascial and muscular interface. For superficial dermal margin optimisation before final closure (averaging 8 to 12 cm in length), the application was performed over approximately 4 min using continuous circular back-and-forth movements along the wound edges until immediate primary tissue coadaptation was macroscopically observed. Consequently, total cumulative NTP exposure per patient averaged 22–26 min per surgical session, delivered under the constant regulated helium flow and radiofrequency parameters described above.

A handheld infrared thermographic scanner (DermaTemp^®^ DT-1001RS; Exergen Corp., Watertown, MA, USA) was used to measure temperature during the interventions. This instrument has a clinical accuracy of ±0.1 °C. This device provides non-contact spot measurements at discrete time points. Because the probe was displaced continuously, measuring the exact point of application was not technically feasible; surface temperature was therefore recorded intermittently at predefined intraoperative intervals at baseline and during application, at sites immediately adjacent to the treated area, including the deep thyroid bed and the vicinity of the recurrent laryngeal nerve. All recorded readings remained between 28 and 30 °C, well below the predefined safety threshold of 35 °C, and no reading approached that threshold. These measurements are reported as supportive procedural monitoring rather than standalone evidence of thermal safety; the principal safety evidence comes from the clinical, biochemical, and histopathological outcomes reported below.

### 2.4. Safety Assessment, Monitoring, and Adverse Event Recording

To maximise clinical reproducibility and avoid biases from segmented timing, the researchers structured the NTP application as a unified, continuous procedural workflow. They carried out this protocol consecutively across all 30 patients to evaluate operational safety and the tissue-handling characteristics of the surgical bed, while safely accommodating the cohort’s natural pathological variability:

- Healing dynamics and superficial tissues: Strict monitoring of surgical wound integrity to rule out incisional dehiscence, seroma formation, subcutaneous fluid collections, or signs of hypertrophic inflammation.
- Postoperative pain control: Systematic quantification of cervical pain intensity using the visual analogue scale (VAS) at each follow-up interval, assessing the patient’s comfort profile and ruling out neuropathic pain or procedure-associated hyperalgesia.
- Functional and neurovascular integrity: Systematic verification of recurrent laryngeal nerve function via clinical assessment of phonation and vocal cord mobility, alongside confirmation of the absence of paresis or permanent dysfunction.
- Deep tissue safety: Confirmation of the absolute absence of iatrogenic thermal injuries, tissue necrosis, surgical bed infections, or obstructive cervical haematomas.

The team meticulously recorded, graded, and classified any local or systemic adverse event according to standardised international surgical criteria and recognised toxicity scales. This rigorous longitudinal follow-up framework ensured complete traceability of the tissue response. It validated NTP’s biocompatibility and early postoperative safety profile in the context of biomedical innovation in thyroid surgery.

### 2.5. Statistical Analysis

Data were processed using SPSS v.26 software (IBM Corp., Armonk, NY, USA). Qualitative variables are expressed as absolute frequencies and percentages. We formally assessed the distribution of quantitative variables using the Shapiro–Wilk test. Because the data were non-normal, we presented all quantitative variables as medians and interquartile ranges (IQR), omitting arithmetic means to avoid methodological interpretation bias.

To address the limitations of a single-arm design, surgical safety was evaluated by reporting each adverse event individually using absolute frequencies, percentages, and exact 95% confidence intervals for the observed incidence. Historical morbidity rates reported internationally for conventional thyroidectomy were included solely as descriptive references [5,31] and were not used as formal statistical comparators. VSS and POSAS scores were also reported descriptively as medians and interquartile ranges.

### 2.6. Operational Framework and Study Design

The project was conducted entirely at the ISSEMYM Medical Centre Toluca. In accordance with current institutional regulations governing advanced surgical procedures and the submission of resected specimens, specialised clinical follow-up covered the immediate, short-term, and medium-term perioperative periods (up to 14 days). The study design was strategically focused on a comprehensive evaluation of intraoperative safety, the operational integration of the NTP workflow, and early histopathological analysis of the surgical bed, ensuring rigorous methodological traceability consistent with the centre’s high-speciality standards of care.

### 2.7. Tissue Integrity and Safety Assessment

To corroborate clinical safety and the absence of thermal iatrogenic alterations from the intervention, we established safety through continuous intraoperative monitoring and postoperative clinical tracking across the 30 patients (*n* = 30). Resected thyroid tissue specimens (including total and partial thyroid resections excised before NTP application) were processed exclusively for routine diagnostic pathology of the underlying thyroid condition. Because directed biopsies of the surgical bed are not part of standard clinical workflows for thyroidectomy, tissue safety assessment relied on rigorous intraoperative biophysical monitoring, including non-contact infrared thermographic scanner, combined with comprehensive macroscopic and functional tracking of the surgical bed throughout the post-operative recovery period.

## 3. Results

The following results are presented as descriptive observations to assess the feasibility, procedural safety, and technical performance of the NTP system. Given the exploratory nature of this pilot study, all statistical tests are descriptive benchmarks to contextualise our findings, not formal hypothesis tests of clinical efficacy.

### 3.1. Demographic Profile and Clinical Characterisation of the Sample

The study cohort comprised 30 adult patients, aged 25–75 years (median age: 41 years; IQR: 37–46 years). To capture the clinical spectrum of thyroid pathology, the sample was evenly distributed between total thyroidectomy (15 patients, 50.0%) and conservative or partial resections, including hemithyroidectomy, subtotal thyroidectomy, and lobectomy (15 patients, 50.0%).

Operationally, the team achieved 100% technical feasibility in integrating the NTP system into the standard operating room workflow. The application protocol proceeded uninterrupted across all 30 procedures, with no device-related power fluctuations, thermal anomalies, or electromechanical reactor failures, confirming the delivery system’s high reliability and ergonomic adaptability within a sterile surgical environment. Table 1 summarises the cohort’s consolidated quantitative safety and healing outcomes.

### 3.2. Surgical Safety, Haemostasis, and Tissue Healing

Consistent with the exploratory design of this pilot cohort (*n* = 30), we evaluated adverse events individually and reported point estimates with exact confidence intervals. Historical rates from international multi-centre registries are provided strictly as descriptive benchmarks [5]. The observed clinical safety findings are detailed across the following domains:

- Incidence of haematomas and haemostatic capacity: No cases of obstructive haematomas were recorded (0/30, 0.0%). Direct NTP application to the thyroid surgical bed immediately following excision coincided with a safe operative profile, with conventional primary haemostatic methods (such as clips and standard ligation) serving as the primary mechanism for vessel control. The immediate perioperative window showed no active haemorrhage, delayed oozing, or clinically detectable fluid collections, and NTP application did not coincide with any obvious bleeding-related safety signals.
- Neurovascular and parathyroid preservation: One hundred per cent of patients maintained normal phonatory function during hospitalisation; postoperative serial laryngoscopic evaluations confirmed symmetrical bilateral vocal cord mobility in all cases. Furthermore, no acute postoperative hypocalcaemia was observed (0/30, 0.0%), with serum calcium levels remaining tightly clustered within the physiological baseline range throughout the intraoperative and early postoperative periods.
- Perioperative aesthetic evolution and early incisional healing: We systematically assessed wound morphology and tissue integrity across standardised postoperative milestones (24 h, 3, 7, and 14 days). Clinical monitoring showed primary epithelial coadaptation and functional wound closure as early as the first 24 h. Subsequent outpatient tracking confirmed an advanced, normotrophic tissue recovery course across the entire cohort (*n* = 30), culminating in complete structural closure by the second week with minimal perilesional erythema, accompanied by favourable early healing metric scores reported descriptively (median VSS: 1.2, IQR: 1.0–2.0; median POSAS overall opinion: 1.1, IQR: 1.0–1.5) to characterise early incisional appearance. Furthermore, no superficial local complications, such as dehiscence, dermal flap necrosis, seromas, or persistent inflammatory reactions, were documented during follow-up.
- Postoperative discharge and recovery timeline: Capitalising on this early tissue stability, most patients met institutional criteria for safe discharge on the first postoperative day (POD1). Although same-day or overnight discharge has become increasingly common following uncomplicated thyroidectomy across various modern international centres, local institutional pathways traditionally entail a 48 h hospital stay. While the absence of a parallel control group precludes a formal comparative quantification, this observed early discharge pattern reflects a notable shift relative to historical local practice and is reported as a descriptive finding in this cohort.
- Deep tissue biocompatibility and tolerance: We confirmed macroscopic tolerance of the deep cervical structures to the cumulative NTP application times across the stratified tissue planes. No evidence of thermal or non-thermal aseptic necrosis, tracheal wall alterations, or structural compromise of the major carotid and jugular vessels was documented during the hospitalisation period, attesting to the procedure’s high biological safety margin.

### 3.3. Macroscopic Characterisation and Tissue Interaction of NTP

**Figure 3:**
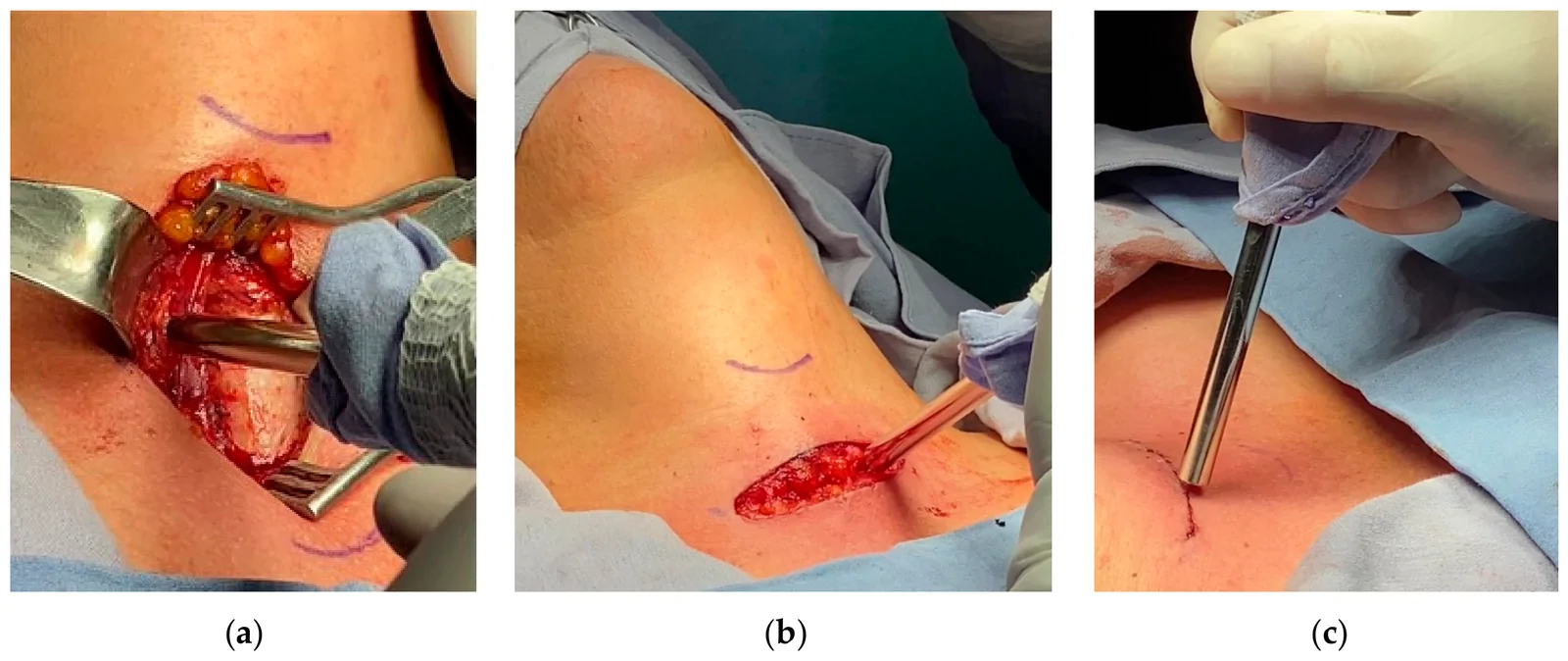
Intraoperative application of NTP in thyroid surgery: (**a**) Treatment in the deep thyroid bed after surgical excision; (**b**) application and modulation in the plane of the exposed pre-thyroid muscles; (**c**) superficial dermal optimisation before final coadaptation of the wound margins.

The iconographic documentation of the protocol provides clear evidence of the biophysical interactions between NTP and the surgical microenvironment across its structured application phases (see Figure 3). Unlike ablative technologies based on conventional heat transfer, the application of NTP at a power density of 0.5 W/cm^2^ macroscopically preserved the structural architecture of all treated tissues.

- Approach to the thyroid bed: Figure 3a illustrates the systematic application of NTP immediately after completing thyroid gland excision. This intervention used a standardised continuous sweep across regions of high anatomical complexity, including the tracheoesophageal groove and the vicinity of the carotid sheath, resulting in a clean, stable bed with no evidence of macroscopic tissue retraction or visible structural damage to adjacent neurovascular elements.
- Modulation in the muscular plane: Figure 3b details the behaviour of the cervical fascia and the prethyroid muscles exposed to the treatment. The elemental muscle tissue showed no charring, desiccation, or thermal debris, consistent with the device’s non-ablative, purely biophysical nature, which maintains local tissue and effluent temperatures within the physiologically biocompatible range (28 to 30 °C).
- Optimisation and dermal closure: The final application was directed to the edges of the surgical wound (Figure 3c), preceding final skin closure. NTP’s interaction with the epidermis coincided with an exceptionally favourable clinical evolution of the superficial tissue, which directly mirrors the superior scores recorded on the perioperative clinical monitoring scales.

A key operational finding of this pilot study is that incorporating the structured NTP application protocol did not prolong overall anaesthetic time or compromise operating room productivity. Because the adjuvant procedure was performed synchronously during the mandatory waiting window for intraoperative frozen-section histopathological analysis (an interval averaging 20 to 30 min at our institution), the protocol efficiently leveraged this inherent logistical delay. This strategic synchronisation integrated NTP therapy without increasing the patient’s cumulative anaesthetic exposure or adding downtime to the surgical workflow.

In summary, the data from this pilot cohort (*n* = 30) demonstrate a robust window of technical feasibility and preliminary perioperative safety for using NTP at a power density of 0.5 W/cm^2^. The intervention successfully treated the surgical bed without evidence of charring, tissue deformation, or immediate functional impairment of surrounding vital structures. These observations underscore the procedure’s high biological safety margin and support the rationale for future randomised controlled trials to formally evaluate the long-term clinical efficacy of this technology in endocrine surgery.

## 4. Discussion

The results of this pilot study indicate that integrating NTP into the thyroidectomy surgical workflow is technically feasible and offers a highly favourable early postoperative safety profile. Unlike conventional electrosurgical or ultrasonic devices, which carry intrinsic risks of lateral thermal dispersion toward the recurrent laryngeal nerve or parathyroid glands [14,32], applying NTP at a power density of 0.5 W/cm^2^ did not cause neurovascular complications in the evaluated cohort (*n* = 30).

This absence of local morbidity coincides with low-temperature biophysical and biochemical dynamics that promote microvascular stability and layered bleeding control without generating macroscopic lateral coagulation necrosis in the deep cervical tissues, remaining well below the critical threshold for thermal tissue damage [32]. A notable clinical observation during prolonged applications (10–12 min) over the exposed pre-thyroid muscle and fascial planes was a subtle, progressive macroscopic alteration in superficial tissue tonality accompanied by a localised matrix coaptation effect. Far from representing overt thermal injury or structural damage, supported by the controlled effluent temperature operating within a safe biological range (28–30 °C), though acknowledging that transient focal thermal micro-effects at the immediate moving interface cannot be entirely excluded by intermittent spot thermography, this phenomenon is attributed to mild, controlled redox-induced protein cross-linking and superficial extracellular matrix modulation mediated by sustained RONS kinetics. Reactive species interacting with interstitial proteins transiently modify optical surface reflection and enhance superficial tissue compaction without compromising deep cellular viability or microvascular architecture.

The biological evolution observed in the skin margins, characterised by exceptionally favourable scores on the VSS (median: 1.2) and POSAS (median: 1.1) scales, is consistent with the mechanisms described for therapies based on RONS [33,34]. At the biophysical level, these outcomes are closely linked to the differentiated molecular kinetics of the RONS generated by the delivery device. During the continuous sweep across structured tissue planes, the NTP interacts with the tissue microenvironment, predominantly delivering short-lived reactive species, such as the hydroxyl radical (^•^OH), nitric oxide (^•^NO), and superoxide anions, alongside controlled micro-oxidation [18,21]. These transient molecules operate at the microvascular scale to accelerate the native haemostatic cascade and promote early tissue coadaptation [35]. Furthermore, controlled RONS delivery has been widely shown in experimental models to modulate dermal wound healing and enhance tissue recovery [36].

When contextualising our recovery timeline, most patients met institutional criteria for discharge on the first postoperative day (POD1). Although early discharge is increasingly common following uncomplicated thyroidectomy in modern international practice, local institutional pathways traditionally entail a 48 h hospital stay. However, in the absence of a contemporaneous parallel control group managed through the same pathway of care, attributing this early discharge timeline directly to the NTP intervention is methodologically unsupported. Consequently, this recovery pattern is reported strictly as a descriptive observation reflecting the early incisional stability documented in this exploratory cohort, rather than a formal proof of comparative superiority [37].

Regarding our safety outcomes, the observed incidence of individual complications (0/30 for haematoma, nerve injury, and hypocalcaemia) aligns favourably with the lower bounds of historical conventional ranges reported in the literature [5]. However, given the single-arm design and the absence of a contemporaneous control group, these historical metrics serve solely as descriptive references rather than formal comparators [23,37]. Therefore, these findings must be interpreted strictly as preliminary indicators of early feasibility and safety, avoiding composite assumptions [38,39].

As an exploratory pilot investigation, the sample size (*n* = 30) was determined based on logistical and preliminary design constraints rather than a formal a priori statistical power calculation, rendering the cohort statistically insufficient to reliably detect uncommon but clinically important complications, such as recurrent laryngeal nerve injury, postoperative haematoma, or hypocalcaemia [26,27]. Furthermore, because the cohort combined total thyroidectomies with conservative or partial resections (hemithyroidectomies and partial procedures), grouping these distinct surgical extents into a single safety analysis complicates granular risk interpretation, given the inherent differences in baseline risks for hypocalcaemia and nerve traction between total and unilateral operations. Consequently, the absence of adverse events in this cohort cannot be interpreted as definitive proof of safety, but strictly as a preliminary descriptive observation.

Regarding intraoperative bleeding control, although the absence of obstructive haematomas (0/30, 0.0%) and direct NTP application to the thyroid surgical bed immediately following excision coincided with a safe operative profile, conventional primary haemostatic methods (such as clips and standard ligation) served as the exclusive mechanism for vessel control; therefore, this observation does not establish primary haemostatic efficacy. Furthermore, this study did not record granular quantitative haemostatic metrics, such as estimated intraoperative blood loss, precise time-to-haemostasis, or exact counts of treated bleeding points, limiting the ability to substantiate haemostatic efficacy quantitatively. Instead, the current data support exclusively that NTP application did not coincide with any obvious bleeding-related safety signal or complication in this cohort, leaving standard surgical haemostasis completely uncompromised. Moreover, because contemporary benchmarks cannot fully substitute for a parallel randomised comparator protocol, historical or literature-derived baselines remain subject to unmeasured biases in surgical expertise and perioperative protocols; hence, causal conclusions remain limited [40,41].

Additionally, clinical follow-up was restricted to the immediate perioperative and early post-discharge recovery windows, and the assessment of recurrent laryngeal nerve and parathyroid integrity relied on established clinical surrogates (serial laryngoscopy and corrected total serum calcium). Consequently, the absence of intraoperative electromyographic monitoring and serial intact parathyroid hormone assays is a recognised technical limitation for detecting subclinical or transient functional impairments [42,43].

Finally, this study has several limitations that warrant acknowledgement. Primarily, the 14-day postoperative observation window, while appropriate for assessing early wound coaptation and acute tissue responses to NTP, precludes definitive medium- or long-term safety evaluations. Consequently, the VSS and POSAS scores reported herein were utilised strictly to quantify and describe the early incisional appearance and primary epithelial healing profile up to two weeks post-surgery, rather than as a definitive assessment of long-term scar maturation, pliability, or final aesthetic outcomes. Future longitudinal studies with extended follow-up intervals are warranted to evaluate the durability of these incisional recovery markers over time.

### Future Directions

The safety and logistical feasibility findings derived from this initial series provide a strong foundation for designing a controlled, randomised, multicentre clinical trial. Building on the infrared thermography monitoring performed in this pilot cohort, the next phase should incorporate continuous infrared thermography to characterise the thermal profile of the surgical bed in real time, alongside a parallel control group receiving standard electrosurgical modalities and objective functional monitoring via intraoperative electromyography, to generate the high-level inferential evidence needed to standardise the surgical technique.

## 5. Conclusions

This pilot study shows that integrating non-thermal plasma (NTP) into the thyroidectomy surgical workflow is technically feasible, logistically efficient, and demonstrates a favourable early postoperative safety profile. The data indicate that NTP application at a power density of 0.5 W/cm^2^ is safely compatible with standard surgical management and supports early primary incisional healing, with no evidence of overt thermal damage or macroscopic neurovascular complications in the deep cervical surgical bed. Furthermore, the absence of major morbidity in this cohort, combined with favourable early healing metrics and clinical-biophysical evidence of tissue integrity, supports the intervention’s biological safety margin. This initial evidence validates the NTP system as a reproducible surgical adjunct and establishes a robust clinical and methodological basis for continued investigation in endocrine practice.

## Figures and Tables

**Table 1:** Descriptive analysis of perioperative surgical safety and intraoperative biophysical monitoring in the group treated with NTP (*n* = 30).

Study Variable	Quantitative Parameter/Observed Incidence	International Historical Reference (Conventional Technique)
Population age	Median: 41 years; Interquartile range: 37–46	N/A
Acute obstructive haematoma	0/30, 0.0% 95% CI: 0.0–11.6%)	0.45–4.2%
Recurrent laryngeal nerve injury	0/30, 0.0% (95% CI: 0.0–11.6%)	1.0–3.5%
Acute postoperative hypocalcaemia	0/30, 0.0% (95% CI: 0.0–11.6%)	1.0–2.0%
Surgical-site infection	0/30, 0.0% (95% CI: 0.0–11.6%))	1.0–2.0%
VSS (14-day early healing)	Median: 1.2 (IQR: 1.0–2.0)	Descriptive assessment (Early incisional appearance)
POSAS (14-day early healing)	Median: 1.1 (IQR: 1.0–1.5)	Descriptive assessment (Early incisional appearance)

## Data Availability

The data presented in this study are available on request from the corresponding author due to ethical restrictions.

## Abbreviations

The following abbreviations are used in this manuscript:

NTP	Non-Thermal Plasma
RONS	Reactive Oxygen and Nitrogen Species
CAP	Cold Atmospheric Plasma
INR	International Normalised Ratio
LPM	Litres Per Minute
RF	Radiofrequency
VSS	Vancouver Scar Scale
POSAS	Patient and Observer Scar Assessment Scale
IQR	Interquartile Ranges
